# Complete Genome Sequences of Two Isolates of *Canis familiaris* Oral Papillomavirus from South Africa

**DOI:** 10.1128/genomeA.01006-16

**Published:** 2016-12-08

**Authors:** Guy L. Regnard, Nwaxigombe M. Baloyi, Leonard R. Bracher, Inga I. Hitzeroth, Edward P. Rybicki

**Affiliations:** a Biopharming Research Unit, Department of Molecular and Cell Biology, University of Cape Town, Rondebosch, South Africa; b Milner Road Veterinary Clinic, Cape Town, South Africa; c Institute of Infectious Disease and Molecular Medicine, Faculty of Health Sciences, University of Cape Town, Cape Town, South Africa

## Abstract

*Canis familiaris* oral papillomavirus, formerly canine oral papillomavirus, is a causative agent of the self-resolving canine oral papillomatosis and was first described in 1994. This is the first report of two full-length genome sequences described in South Africa and indicates the highly conserved nature of *Canis familiaris* oral papillomavirus.

## GENOME ANNOUNCEMENT

*Canis familiaris* oral papillomavirus (CPV1), formerly canine oral papillomavirus, is the causative agent of the self-resolving canine oral papillomatosis (1–3). Thus far, only CPV type 1 (CPV1) and CPV13 have been isolated from canine oral papillomas (4). The circular double-stranded DNA (dsDNA) virus belongs to the genus *Lambda* from the *Papillomaviridae* family (1). A full-length genome of CPV1 was first described in 1994 from a strain used in the preparation of a live vaccine against CPV (5). This sequence is identical to the one deposited at the Papillomavirus Episteme site (http://pave.niaid.nih.gov) (6).

In this study, a Dobermann and Siberian husky, both 9 months old and raised separately, were diagnosed in September 2011 with oral papillomatosis. Multiple lesions were identified on the buccal lips and gums. These were approximately 3 to 5 mm in diameter and height and typically fimbriated. Samples were collected from lesions and stored at 4°C. Total DNA was extracted using a DNeasy blood and tissue kit (Qiagen, DE) and prepared as per manufacturer’s instructions for the purification of total DNA from nucleated animal blood cells (spin column protocol). Two overlapping 5-kb sections of the genome were amplified using Phusion high-fidelity DNA polymerase (New England BioLabs, USA) and primer sets 5′-TCGGTGAGAGATCTTTGC-3′ and 5′-GACTCTTGGTCAGATAAGC-3′, and 5′-ACCTGCTCTTTCATCATAC-3′ and 5′-TGCGGACTAAGGCTACAC-3′. The thermocycling conditions were an initial denaturation at 98°C for 30 s, followed by 25 cycles of 10 s at 98°C, 15 s at 51°C, and 150 s at 72°C, and a final elongation step at 72°C for 10 min. The amplified DNA was cloned using the CloneJET PCR cloning kit (Thermo Fisher Scientific, USA), as per manufacturer’s instructions, and the sequence of the DNA was determined using primer walking (Macrogen, Republic of Korea). The sequences were assembled using DNAMAN (version 5.2.9; Lynnon BioSoft).

The full-length genome sequences were compared to the reference genome (Papillomavirus Episteme). The two sequences were 8,607 bp and 8,606 bp, and seven open reading frames were identified for each, containing early genes E1, E2, E4, E6, and E7 and the late genes L1 and L2. No E5 has been reported for CPV1 (7) and was not identified in this study. The two sequences differed by two nucleotides, with one synonymous mutation within the L1 gene and a deletion within the intervening sequence between the E2 and L2 genes. The sequences shared greater than 99.9% identity with the reference sequence and 99 to 100% identity with CPV1 sequences in GenBank.

This is the first report of full-length genome sequences of CPV1 described in South Africa. The report indicates the highly conserved nature of CPV1 in comparison to the reference sequence described 16 years earlier, from samples gathered thousands of kilometers away, with no conceivable connection between the incidences.

### Accession number(s).

The complete genome sequences for *Canis familiaris* oral papillomavirus have been deposited in GenBank under the accession numbers KX587460 and KX587461.
